# Barriers and facilitators in the delivery of a proportionate universal parenting program model (E-SEE Steps) in community family services

**DOI:** 10.1371/journal.pone.0265946

**Published:** 2022-06-13

**Authors:** Vashti Berry, Siobhan B. Mitchell, Sarah Blower, Karen Whittaker, Kath Wilkinson, Sinead McGilloway, Amanda Mason-Jones, Rachel Margaret Carr, Tracey Bywater

**Affiliations:** 1 College of Medicine and Health, University of Exeter, Exeter, Devon, United Kingdom; 2 Department of Health Sciences, University of York, York, North Yorkshire, United Kingdom; 3 School of Nursing, University of Central Lancashire, Preston, Lancashire, United Kingdom; 4 Centre for Mental Health and Community Research, Department of Psychology and Social Sciences Institute, Maynooth University, Maynooth, Co Kildare, Republic of Ireland; Flinders University, AUSTRALIA

## Abstract

**Background:**

A proportionate universal (PU) approach to early years’ service provision has been advocated to improve children’s health and development and to reduce health inequality, by ensuring that services provide timely and high-quality parenting support commensurate with need. Process-oriented research is critical to examine the factors that contribute to, or hinder, the effective delivery/implementation of such a model in community-based family services. This study aimed to assess the delivery, acceptability and feasibility of a new PU parenting intervention model (called E-SEE Steps), using the Incredible Years® (IY) parent program, when delivered by trained health/family service staff in three “steps”—one universal step (the IY Babies Book), and two targeted steps (group-based IY Infant and Toddler programs).

**Methods:**

An embedded mixed-methods process evaluation within a pragmatic parallel two-arm, assessor blinded, randomized controlled trial was conducted in community services in four local authorities in England. The process evaluation used qualitative data gathered via interviews and focus groups with intervention arm parents who were offered the targeted steps (n = 29), practitioners (n = 50), service managers (n = 7) and IY program mentors (n = 3). This was supplemented by quantitative data collected using group leader pre-training (n = 50) and post-delivery (n = 39) questionnaires, and research notes of service design decisions.

**Results:**

The E-SEE Steps model was acceptable to most parents, particularly when it was accompanied by engagement strategies that supported attendance, such as providing childcare. Practitioners also highlighted the positive development opportunities provided by the IY training and supervision. However, participant views did not support the provision of the IY Babies book as a standalone universal component, and there were barriers to eligible parents—particularly those with low mood—taking up the targeted programs. Service providers struggled to align the PU model with their commissioned service contracts and with their staff capacity to engage appropriate parents, including tackling common barriers to attendance.

**Conclusions:**

Despite general enthusiasm and support for delivering high-quality parenting programs in community services in the England, several barriers exist to successfully delivering IY in a proportionate universal model within current services/systems.

## Introduction

The earliest years in a child’s life are a critical period in their development, during which time responsive and empathic parenting can promote positive outcomes [1]. In 2010, the Marmot Review concluded that good quality services in the early years, including parenting support, can have enduring effects on health, but that action was needed across the system to reduce inequalities in terms of physical and mental health and the development of appropriate cognitive and social skills [2]. Marmot et al. suggested that these actions must be universal but with a scale and intensity relative to the level of disadvantage—an approach they described as “proportionate universalism” [2 p15].

The principle of proportionate universalism (PU) has gained both support and objection since Marmot’s 2010 review, largely due to tensions inherent between universalism and targeting policies [3, 4]. Carey and colleagues argue that while the principle is theoretically appealing, its application in practice has been limited by a lack of guidance for policy makers and practitioners wishing to implement a PU approach [5]. Furthermore, there have been different interpretations of what a ‘proportionate’ response is, or should be, in practice; some commentators [e.g., 6] favor broad universal provision while others [e.g., 7] advocate for better targeting of, and increased provision for, disadvantaged families. A recent systematic review of early childhood parenting interventions, aiming to reduce social inequalities in children’s health and development, found only 23 interventions across Europe [8]. Of these, only two met criteria to be considered PU models, with their intervention levels tailored according to need; the remaining programs were all categorized as targeted provision, aimed at specific populations, e.g., economically disadvantaged families or those living in deprived neighborhoods. There is a need for more descriptive and empirical studies to explore how PU models work in practice.

This paper details process evaluation findings of a PU model of parenting support for the parents of children under two years, called **E**nhancing **S**ocial-**E**motional Health and Wellbeing in the **E**arly Years (E-SEE Steps), for the parents of children under two years. The intervention model and findings on its clinical and cost-effectiveness are detailed in our companion trial outcome paper [9] (full protocol available at: https://www.dev.fundingawards.nihr.ac.uk/award/13/93/10). The trial demonstrated no positive effect of the PU intervention on either parent depression or child social emotional wellbeing, when compared to the control arm, and it was concluded that changes to the parenting program and the service delivery system may be required. The present study builds on these findings to explore the barriers and facilitators of a PU model in practice.

### E-SEE Steps

The constituent programs of the E-SEE Steps model are taken from a parenting series called the Incredible Years® (IY), based on social learning and attachment theory [10, 11] and designed to promote social and emotional health and wellbeing by improving parent-child interactions and enhancing attachment and parent sensitivity. E-SEE Steps can be considered a PU model because it comprises two IY programs (IY-I and IY-T) delivered in three steps—one universal dose, and two subsequent targeted/indicated steps determined by need, delivered as the children age (**S1 Fig**). Program content is summarized in **S1 Table**. The universal dose (the IY-B book) is suitable for all parents of babies under 12 months and was posted to all intervention parents, after baseline and randomization, to increase awareness of their babies’ socioemotional needs. Revisions to the book were made following our external pilot trial [12] and the definitive trial tested Edition 2 [13]. Parents were eligible for the targeted steps of the PU model—the IY-I or IY-T programs—if at follow-up 1 (2 months post baseline) or 2 (9 months post baseline), respectively, they obtained ‘mildly depressed’ or higher scores on the Patient Health Questionnaire (PHQ)-9 (a 9-item self-administered measure of depressive disorder) [14], and/or if their child scored in the ‘monitoring zone or above’ on the Ages & Stages Questionnaire: Social-Emotional, Second Edition (ASQ:SE-2) [15] (a parent-completed measure of their child’s social emotional development). The IY-I and IY-T targeted group sessions were delivered weekly in collaborative two-hour discussion sessions, plus exercises to practice at home.

While there is substantial evidence to support the use of parenting programs (and IY in particular) with parents of children aged 3–12 years in order to prevent and manage emotional and behavioral difficulties, the evidence is equivocal for younger children [e.g., 16–20]. In the UK, the IY Infant (IY-I) program has shown promise of effectiveness in a comparison study [21] although the effectiveness of the IY Toddler (IY-T) program has yet to be established [22]. In addition, while prevention and early intervention programs are more likely to achieve positive outcomes when implemented with fidelity [23] and when parental engagement is high [24], less is known about the implementation barriers/facilitators, processes and context of parenting interventions for infants and toddlers. Only a minority of parents who could benefit from such evidence-based interventions receive them in practice [25, 26]. A recent Individual Participant Data meta-analysis found no evidence for differential effects of IY by social disadvantage [27], suggesting it can be suitably tailored to need and there is evidence to suggest that severely depressed parents of children with serious conduct problems are likely to benefit most from the intervention [28].

More process-focused research is now needed to examine how these interventions can be embedded within real world community services for children and families, and to understand the individual, organizational and system-level factors that contribute to or hinder effective delivery [29–31]. The present study seeks to advance this understanding by presenting findings from an embedded process study, as part of a multi-site randomized controlled trial of a PU intervention delivered by public services to parents of children under two years.

## Materials and methods

### Research questions

Our paired trial outcome paper [9] reported on the effectiveness and cost-effectiveness findings of E-SEE Steps, the uptake of the E-SEE Steps intervention by parents and the fidelity of IY program delivery by public sector services. It found a low rate of conversion from parents’ eligibility to take up of the parenting programs (50% for IY-I and 21% for IY-T), low program completion rates (45% for IY-I and 43% for IY-T) and varying levels of adherence by site and group/practitioner, with a mean fidelity rate of 64% for IY-I and 74% for IY-T. However, there were high levels of parent satisfaction with program content and process (3.4 and 3.7, out of 4, for IY-I and IY-T respectively). This process evaluation paper builds on, and elucidates those findings via two pre-defined research questions:

1. Can a multi-agency service deliver the IY program in a PU model, and what are the barriers to and facilitators of successful delivery?
2. How acceptable and feasible is delivery of E-SEE Steps model for key intervention stakeholders?

### Service design

The research team supported sites to tailor E-SEE Steps to their context. This included a series of in-person workshops, videos and information sheets for practitioners to identify and refer parents to the trial, and tailored service design support to set up the targeted group-based programs (IY-I and IY-T) in their areas. This included supporting sites to identify delivery locations to maximize access, select group leaders, employ appropriate parent engagement strategies, and make use of program fidelity data.

A detailed 64-page Service Design Manual for the E-SEE Steps intervention (freely accessible from: https://www.york.ac.uk/healthsciences/research/public-health/projects/e-see-trial/) was developed to provide more detail on the theoretical foundations of the intervention, including a logic model, and detailed implementation guidance. Implementation briefings were also created for each individual site. Service design decisions and key challenges in each of the four sites were systematically logged by the research team and are described below.

E-SEE-Steps was designed to be delivered by a combination of Early Years Children’s Services and/or Public Health Nursing staff, trained by accredited IY mentors (and supervised regularly) to deliver IY as part of the trial. However, the intervention was led by a single agency in two of the four sites, reflecting the variable nature of existing partnerships and cross-agency working arrangements for service delivery. The IY developer recommends that group leaders have prior training in child development and cognitive social learning theory, as well as experience with young children, parenting skills, and family interactions. Health visitors, providing the universal new birth home visit (10–14 days after birth), were the main agents for identifying and inviting mothers to join the study. A small minority of parents were recruited via community children’s services or self-referral. Researchers followed-up referrals to complete the recruitment process and sent a copy of the IY-B book (universal step) to all parents who had been randomized to the intervention.

A key challenge for sites was the timing of the invitation—study and intervention information had to compete for parents’ attention with a range of health education material already required as part of the new birth visit service. Moreover, in real world delivery, professionals delivering a PU service would be continually assessing families’ needs and timing their offers of help to match needs and receptivity. E-SEE Steps was not personalized in this way; constrained by the trial design, the model provided targeted support at set points in the child’s development corresponding with the age range of the IY-I and IY-T programs.

Supervision for leaders delivering the group-based programs was provided by accredited IY mentors remotely, on a fortnightly basis during delivery. Due to difficulties with data protection and organizational firewalls, the planned sharing of group video footage with mentors for review of practice was not possible for most of the groups, and supervision sessions relied on leaders’ self-assessments. Some IY group leaders (four groups across two sites) managed to arrange face-to-face sessions with mentors to achieve this review.

In line with IY program guidance, the E-SEE Steps model emphasizes the importance of sites employing engagement strategies to facilitate access to parenting support. Sites differed in their ability to fund and/or provide the recommended support, including introductory home visits, provision of childcare, transport support, preparation and engagement time for leaders. Suggested leader preparation also included undertaking dry runs of delivery to increase familiarity with the materials and processes but only one site managed to achieve this. Targeted E-SEE Steps were planned as four IY-I and two IY-T groups in each site, however, all four trial sites delivered only two IY-I groups each; two sites delivered two IY-T groups and two sites delivered only one. Health professionals’ time for training and delivery was covered by NHS excess treatment costs but there was no budget support for Children’s Services’ staff and participation was contingent on existing resources.

The control group/arm received services as usual (SAU); IY-B, IY-I and IY-T were not offered as SAU in trial delivery sites. As part of the service design work, a profile of SAU in each site was created in order to capture availability of childcare and parenting services to both intervention and control families (**S2 Table**). This revealed a wide range of support available to families from post-natal support and healthy weight and nutrition, to behavior management, early learning and development, and support for children with additional needs. While several parenting programs were on offer across sites (e.g., Triple-P, Strengthening Families and the Solihull Approach) the service receipt data shows that less than five parents in each arm accessed these parenting support programs.

### Sampling and recruitment

**Fig 1:**
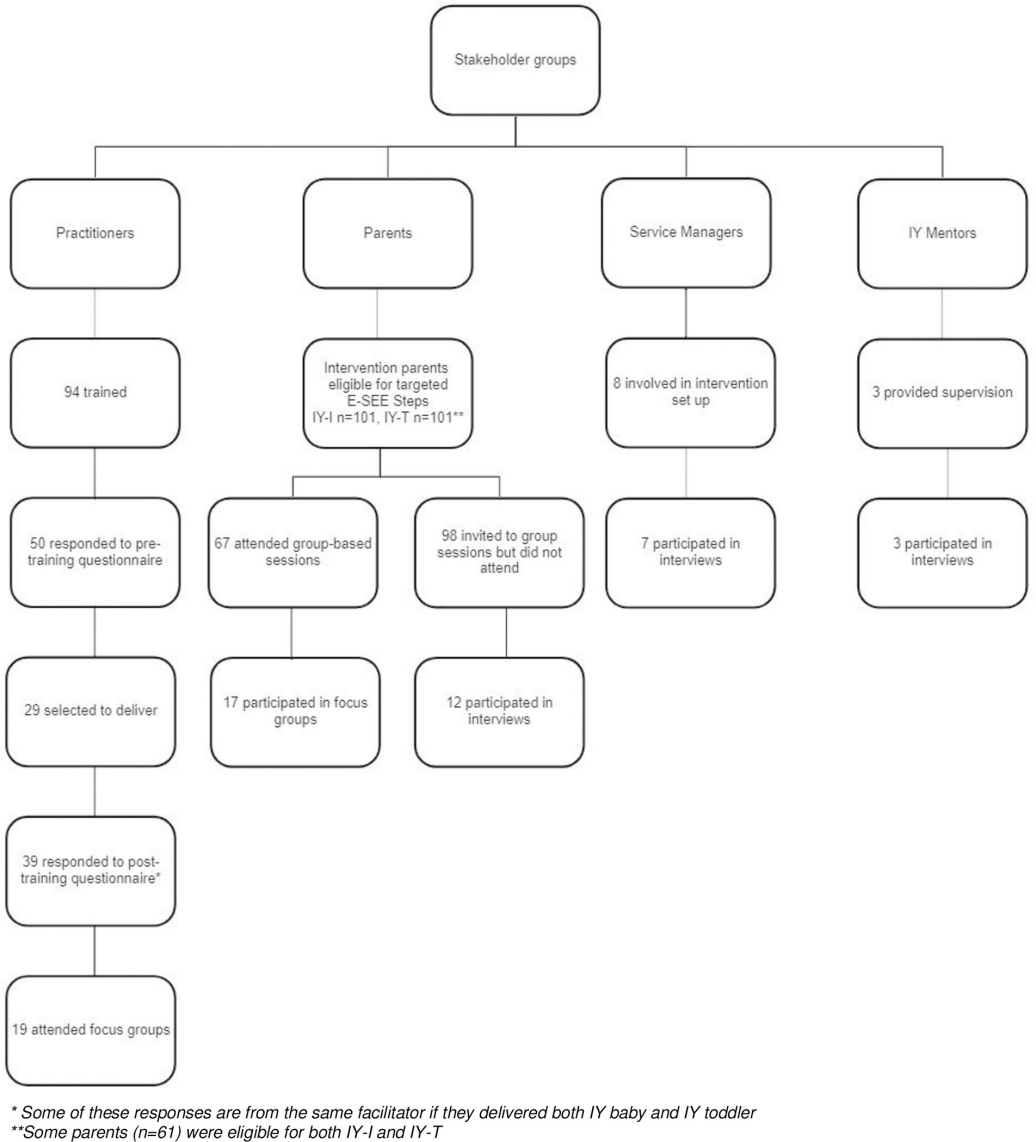
Flowchart of process evaluation participants.

In addition to the service design summarized above, the process evaluation collected data from three stakeholder groups across the four trial sites, as outlined below (see Fig 1).

#### IY group leaders

Ninety-four practitioners (93 female, 1 male) were selected by site managers to be trained as prospective group leaders, and 29 (31%) of whom went on to deliver the IY-I and IY-T groups. All of the 29 group leaders who delivered the IY parenting programs were invited to participate in research focus groups; 19 agreed to participate.

#### Service managers and IY mentors

Seven of the eight service managers (2 from each site) who were involved in intervention set-up participated in research interviews. All IY mentors (n = 3) were invited and all participated in individual interviews.

#### Parents

A total of 17 out of 67 (25%) intervention parents and their co-parents, who had attended at least one of the group-based sessions, agreed to take part in a focus group on completion of outcome data collection. Purposive sampling was also used to select a group of 12 parents for interview, who were eligible for the targeted IY-I/IY-T groups but who declined/did not attend the sessions.

### Ethical considerations

This study was approved by National Health Service (NHS) North Wales Research Ethics Committee (REC) 5, Bangor on 22nd May 2015 (REC Reference 15/WA/0178, IRAS 173946), and by Departmental Ethics Committee, University of York on 10th August 2015 (Reference number FC15/03). All participants gave written informed consent.

### Data collection

Parents were consented into the process evaluation as part of the intervention trial consent procedures [see full protocol at: https://www.dev.fundingawards.nihr.ac.uk/award/13/93/10]. The process evaluation team (KW, SM, VB) obtained written informed consent from IY group leaders, service managers and IY mentors independently using process evaluation information sheets and obtained informed consent. All evaluation information sheets and consent forms for practitioners and service managers/mentors as well as pre-training and post-delivery online questionnaires can be downloaded from https://www.york.ac.uk/healthsciences/research/public-health/projects/e-see-trial/.

#### Quantitative measures

IY group leader data and feedback was obtained using two specially designed online questionnaires. Fifty practitioners completed the pre-training questionnaire, which gathered information on demographics, qualifications and experience of working with families. Group leaders who delivered the targeted intervention steps also completed a post-intervention delivery questionnaire (n = 39); this was used to supplement the qualitative focus group data on group leaders’ views of delivering the group-based programs by asking questions about organizational support for delivery, e.g., “On a scale of 1–5, how supportive was your organization of practitioners delivering the Incredible Years parenting groups?”

#### Qualitative methods

Eight focus groups and 22 one-to-one interviews were undertaken by four experienced, female researchers (VB, SM, RC and KWh), following the completion of intervention delivery in each site. Four focus groups were conducted with group leaders (n = 19) and four with parents and co-parents who had attended the group-based intervention (n = 17). These were conducted in person by two researchers (one leading, and one note taking) and lasted 90 minutes. Semi-structured topic guides explored: the acceptability and usefulness of the IY-I book as a universal intervention; the acceptability of a proportionate universal model; processes for identification, screening and recruitment; strategies/ approaches for engagement; barriers/facilitators of attendance; and experiences of participation in the groups (see **S1 File**).

Ten semi-structured interviews (lasting approximately 30 minutes each) were conducted in person or by telephone with health and children’s services managers and IY trainers/mentors. These interviews explored participants’ views on delivering IY as a proportionate universal model, accommodations/adaptations made, organizational or system-level barriers and facilitators to delivery. Twelve semi-structured telephone interviews (approximately 15–20 minutes each) were also undertaken with non-attending intervention parents to explore why parents did not engage/discontinued engagement. All interviews were audio recorded and transcribed verbatim, checked for accuracy and any identifying comments or observations removed.

#### Quantitative data analysis

The group leader questionnaires were analyzed descriptively in SPSS 25 using means, medians and percentages.

#### Qualitative data analysis

To explore the barriers and facilitators to delivering E-SEE Steps, thematic qualitative analysis was used following a Framework Analysis approach [32]. Interviews were coded around individual, organizational and system level factors influencing implementation. Summaries of each code and themes were created and patterns explored. Coding was progressively refined and themes were developed and agreed through team discussion (VB, SM).

To explore the acceptability and feasibility of E-SEE Steps, thematic analysis was carried out, using a deductive/theoretical approach (with the support of NVivo 11, QSR International, Brisbane). Interviews were read and re-read by SM, and data were coded using a semantic approach [33]. Data were organized to explore patterns in semantic content within and across data sources in relation to the research questions. Coding was progressively refined with the researcher moving between codes, accounts, thematic maps and analytic memos and notes, which were written throughout the process to explore emergent thoughts, ideas, concepts and themes. Themes were reviewed and refined by the process evaluation team (VB, SM), and the significance of the patterns and their broader meanings and implications theorized in relation to the research questions [34]. Results were shared with group leaders and service managers in all sites in site-level video-conference result meetings (each lasting two hours) (in line with COREQ checklist criteria); interpretations drawn reflect a shared understanding of the data [35].

## Results

### 1. Barriers and facilitators to delivering E-SEE Steps

Group leaders, service managers and IY mentors were asked about the barriers to, and facilitators of, delivering E-SEE Steps with adherence. A total of three overarching themes (and five sub-themes therein) were identified, relating to individual, organizational and system level factors (see Table 1). (see **S3 Table** for supporting quotations). Each of these themes is discussed below alongside, where relevant, the questionnaire findings. Individual practitioners’ qualifications, experience and confidence in delivering parenting programs varied, which may have influenced their delivery of IY-I and IY-T programs (see **S4 Table**).

**Table 1:** Barriers and facilitators: Themes and sub-themes.

1. Individual level factors
a. Connection and relevance
2. Organizational level factors
a. Engagement processes
b. The IY program
c. Supervision and mentorship
3. System/service level factors
a. Support for implementation

#### Theme 1: Individual level factors

*Connection and relevance*. The IY program was seen as a critical facilitator of connected and personalized parenting support. The importance of building rapport with parents at an early stage, ideally pre-delivery, was highlighted by group leaders and was noted as particularly important in engaging parents who were struggling with mental health or other challenges.

Building rapport with parents was made more challenging by capacity and the short period of time allotted for engagement. Rapport was particularly important in cases where the parent was anxious or there were other challenges being faced:

*“[Name] knew both of the people that did end up coming*, *and like you said, they are unlikely to attend, that demographic are unlikely to attend group, and I think they did partly because they knew she knew them…That makes a difference.”*

(Leader)

IY mentors also acknowledged the difference that good rapport can make:

*“…a lot of them had good relationships already with either the health visitor or some of the team*, *and that made a big difference.”*

(IY mentor 2)

In addition to building rapport, group leaders’ understanding of individuals’ goals and needs, “knowing your group” enabled sensitive facilitation, personalization of the manual and the ability to better manage group dynamics:

*“… it is that real personalization*. *That makes sense doesn’t it*? *If it is pertinent to you*, *you are going to be engaged in it*, *you are going to be interested*. *You are going to feel like you are getting a lot out of it whereas if it is not*, *then not necessarily*.*”* (Leader)

#### Theme 2: Organizational level factors

*Engagement processes*. Group leaders and service managers described the challenges of recruiting and engaging parents. Reported barriers were related to the processes of service engagement, specifically the timing and relevance of the offer i.e., identifying the right parents at the right time, and “selling” the offer, in particular the clarity of the offer in terms of what the program involved, and where and when it would take place. For example, group leaders highlighted the importance of the gap between initial contact with a parent and the group starting in terms of engaging parents

*“…things change from that initial conversation*, *that point where they were recruited at the beginning, maybe that mood has improved, maybe they weren’t struggling so much with baby.”*

(Leader)

In terms of communicating clearly to parents what the group would offer, group leaders reflected on a lack of certainty around factors such as creche provision making this more challenging:

*“The kind of thing that came up when I was contacting parents for toddler group was that we didn’t have the crèche and we weren’t sure if we were going to have a crèche…So that was kind of like yeah*, *I can come if that’s…there but if not, definitely not.”*

(Leader)

Early identification and the opportunity to build relationships and attune to parents’ needs are needed to facilitate engagement and the delivery of this model:

*“It’s really about maintaining that contact and identifying when is the right time because for a lot of parents you don’t walk past your front door for quite some time when you’ve had a newborn baby*. *You’re getting your routine in place…*”

(Service manager)

*The IY program*. There was evidence of some practitioner resistance to a few of the core IY processes/activities, such as role play with which, arguably, not everyone would feel comfortable:

*“… [they] were very resistant, to role play… They were resistant in the training and they*, *that moved on and although we spent, I spent more time than anything else I think on…on trying to problem solve that with them.”*

(Mentor)

Adapting the program with fidelity was also not straight-forward; for example, adapting to small group sizes and different ages, and leaders reflected the preparation time needed:

“…*the particular challenge was for us was the babies that we had… trying to tailor and… still deliver the program how it should have been delivered*.*”*

(Leader)

*“Well, it just meant that everything became a group exercise really because if you…You couldn’t do things like split into smaller groups…”*

(Leader)

Existing professional practice was also raised as a potential barrier, with the collaborative (non-didactic) IY approach being considered quite different to the current culture and practice in the service organizations: *“… there’s still much*, *an awful lot of teaching and not that much collaboration going on*. *I think a lot of advice giving may have been given…”* (Mentor).

There was a consensus that an additional booster training session would help to prepare practitioners to better negotiate these challenges but that the timing of this–in relation to delivery–is important to ensure connections are made between learning and practice:

*“I think it’s also the timing*, *like when you actually do your training and like the gap but then you’ve got to up and run it and you’re like, oh my God…Just like a refresher… Yeah. Like a mock session.”*

(Leaders)

*Supervision and mentorship*. Group leader skills, supervision and mentorship were regarded as important factors in supporting program delivery with fidelity. Indeed, this was also reflected in the post-delivery questionnaires (See **S4 Table),** which showed an increase in the number of group leaders who reported feeling confident in delivering a parenting group (42% pre-training cf. 59% post-delivery). However, a significant minority still lacked confidence even after delivering IY-I/IY-T. The group leaders who participated in interviews, described how IY mentor guidance and supervision sessions helped them to grasp the collaborative process and develop their practice:

*“… it has changed her practice*, *it really has, where before she would have stepped in, intervened and offered the advice and guidance, she now knows how to facilitate that conversation to enable parents to reach that understanding and that decision making and empowering them back to be able to do that… She will always say, “It totally changed the practice,” which is really powerful because people can get set in their ways and continue to do what they’ve always done…”*

*(*Service Manager*)*

However, challenges arose from the logistics and (remote) mode of supervision. Group leaders felt that live supervision offered the best support for delivering with fidelity, particularly in terms of reviewing and reflecting on video recordings:

*“Because the Skype supervision… it didn’t work, and you couldn’t unpick your videos, and I think that’s what you need for the Incredible Years… We tried to figure it out, but with governance we couldn’t really do it. And that’s for me, the live supervision, how it should be, and I benefitted from that because it just helps you keep to that true fidelity, doesn’t it?”*

(Leader)

#### Theme 3: System/service level factors

*Support for implementation*. Capacity and managerial pressures could be a barrier to delivery and specifically capacity for planning and preparation, with competing service demands posing an additional challenge to the successful delivery of the program as prescribed. In contrast, adequate support within the system, specifically flexibility and understanding of managers, was found to facilitate fidelity:

*“… we tried to do gold standard again but we couldn’t quite get there… [It] has been challenging because there’s been other demands, and you just never know what’s coming in*. *That’s our problem, things can change from one week to the next and one day to the next…”*

(Service Manager)

*“On my manager’s side she’s kind of just given me free reign to you know manage my time around it and I didn’t feel pressure from her*, *I felt that actually we could just do it and manage it ourselves…”*

(Leader)

Notably, in the post-delivery questionnaires, most IY group leaders (80%) reported that their organizations had been supportive during delivery, ensuring that practitioners had sufficient time, resources and encouragement to deliver programs effectively (See **S4 Table**). Reasons given for lower organizational support ratings included reports that no changes were made to existing caseload to free up time for delivery, not enough time was given to prepare and prioritization of other tasks over delivery.

### 2. Acceptability and feasibility of E-SEE Steps delivery

#### Acceptability

Findings from the fidelity analysis [9] support a good level of satisfaction and general acceptability for attending parents but low conversion between acceptance of a group place and attendance. Qualitative data from parents and group leaders suggest this was likely due to the timing and nature of the offer and mixed acceptability for parents with low mood or depression due to the demands of attendance and program processes. Table 2 presents the four main themes related to the acceptability of E-SEE Steps (see **S3 Table** for supporting quotations).

**Table 2:** Themes and sub-themes related to acceptability of E-SEE Steps.

1. Nature of the intervention offer
a. Receiving and understanding the offer
b. Familiarity
c. Crèche
d. Co-parent attendance
2. Demands of attendance and program processes
3. Utility of the IY-B book
4. Nature of a parenting group

*Theme 1*: *Nature of the intervention offer*. Four sub-themes were identified in relation to the acceptability of the intervention offer: (1) receiving and understanding the offer, (2) familiarity, (3) crèche, and (4) co-parent attendance. Several parents who did receive the offer said they lacked an understanding of what it entailed. They understood that it would be a group of parents but were unsure of what they would be doing in the group and suggested that they would have been more comfortable if they knew more detail about the group and a breakdown of what the sessions involved:

“*I don’t think it was enough information, because I remember starting coming to the groups, and it kind of all had to get explained to me again*. *I didn’t really know what I’d actually joined up for… I don’t really think there was a lot of information given when it was…So you’re like, yeah you’ve been invited to come to this group, but then I actually had no idea what was… what to expect from the group*.”

(Parent)

All key stakeholders perceived familiarity and trust as important to acceptability, with the relational nature of the offer highlighted as critical. Familiarity was important for attendance both in terms of knowing other parents attending the group and in terms of familiarity with IY processes, e.g., having attended IY-I, parents knew what to expect at IY-T. This was particularly the case for non-attending parents and those who felt anxious about attending a group. A local venue and the option to attend with a co-parent or friend were suggested to increase acceptability:

“*Yeah*, *I think that would have helped*, *knowing at least one other person that was going*. *Obviously*, *you don’t know anyone*. *That probably would have been more encouraging*.*”*

(Non-attending parent).

Although crèche provision is known to promote and maintain attendance at parenting groups, for those who chose not to attend it was viewed as an irrelevant resource, either because they did not want to give up the time they had with their child when not at work, or because they felt uncomfortable leaving their child in the crèche:

*I’m not on maternity leave anymore, it meant giving up quite a chunk of time with my kids on the only day I have off with them*… *And I didn’t really understand why they had to be in a separate room*.

(Non-attending parent)

Several parents reported not knowing that they could have had a co-parent attend with them and some of them felt this would have made a difference to their decision to attend:

“…*Better if you could bring somebody with you*…*I know you’re a single mum but if you could bring like your mum with you*…”

(Parent)

“*Just to be*… *to listen to what’s been said and sort of engage with it too because they’re your main caregivers for your child…That’s what you need*.”

(Parent)

*Theme 2*: *Demands of attendance and program processes*. There was a mixed perception of acceptability for parents with low mood or depression due to the demands of attendance and the program processes.

Group leaders questioned the fit between the demands of the IY program and parents with low mood and depression. They suggested that low mood could be a barrier to attendance, with those parents finding it difficult to commit to a group when there is a lot going on at home:

“*I suppose you’ve got that additional barrier with some of the participants that you are targeting*. *That definitely was the case in our last toddler, there was someone whose mental health deteriorated so they couldn’t continue to… mood and baby IY is usually the barrier as well, just the fact that they have got the baby, it is really hard to get out to a group*.”

(Leader)

This was also identified as a barrier by non-attending parents:

“*… the first time when they were babies I couldn’t go because my anxiety manifests as agoraphobia*, *and I couldn’t get… I just couldn’t get out the house*.*”*

(Non-attending parent)

“*Yeah, and I didn’t go only because I was suffering quite bad with anxiety at the time and I didn’t feel comfortable going without my partner but*, *because it was only set times, my partner was at work for most of those days*.”

(Non-attending parent)

There was mixed acceptability around the program processes for group leaders also. Specifically, leaders were cautious about using role play–one of the intervention’s primary processes–which they reported as difficult to successfully engage in for those with low mood:

“*Did low mood prevent any parents from participating…Definitely… One of ours, she came to a little bit towards the end, didn’t she?* …*That’s why we had to be careful about role play, wasn’t it, that sort of woman who shutdown. Also, in the baby group there was a couple that we kept trying to get but they just couldn’t. Because being in a group… for some people’s really difficult as well*.”

(Leaders)

“*Role play was the problem*… *and [name of mentor] asked us to even if they were sat on their seats just to try and get them to interact in some way and when we did she just… just totally went into herself whereas before that we hadn’t, we just let whoever wanted to participate*.”

(Leader)

Although leaders found different ways around this, like demonstrating the role play themselves, they were unsure whether it was feasible to engage with and deliver the program successfully to those struggling with low mood. Group leaders felt that this may have been exacerbated by small group sizes and these parents may have been more comfortable with the demands of IY within a larger group.

In instances where parents who were struggling with low mood did attend and engage with sessions, leaders felt that active engagement between sessions (e.g., phone calls and follow up) were central to ensuring that low mood didn’t become a barrier to attendance:

“…*so in the baby one, the only person we’ve had that’s done both baby and toddler with us, the second week she didn’t come to baby and we gave her a phone call and were chatting to her and she said yeah, I really struggled, I j… couldn’t get out the house this week, I just couldn’t do it*. *So we had a good chat with her… Having that open communication with her and her feeling okay to open up to us, that she was feeling low, that then helped overcome that barrier*.”

(Leader)

*Theme 3*: *Utility of IY baby book*. The use of IY-B book as a universal intervention was intended to provide parents with a physical/close-to-hand educational resource similar to the NHS resources (e.g., “Birth to Five”) that have been digitized/put online in recent years. Parents who were interviewed reported finding the IY-B book difficult to access and largely engaged with it through practitioner signposting–calling into question the utility of book as a standalone resource (the universal level of E-SEE Steps). Use of the books was largely practitioner-led within targeted groups, and some of the difficulties highlighted by parents were intellectual or unfamiliar language (Americanisms). The accessibility of the book was also questioned by parents who said that the size of the book and its language could be quite intimidating and difficult to understand:

“*A lot of it’s intellectual*. *So it’s really hard to understand…So it’s like, what’s that word? Google it…I sort of understood it, only because I’m in childcare. But some of the words were a bit like, oh, you have to know what that is*.”

(Parent)

*Theme 4*: *Nature of a parenting group*. Being part of a group was described as the most valued outcome for parents attending the sessions. The opportunity to get out of the house and to meet other parents with shared experiences and challenges was an attractive part of the group:

“*Just the relationships I built with the people that were running it and the other mums*…*Yeah*, *we were all in it together*…*Yeah*, *and everyone became like friends*, *if you will*.…*And then everyone was interested in each other’s babies and things*, *so I think that’s what helped*. *And everyone knew each other*, *like rather than… it just felt like you were friends rather than just people that were…Yeah*. *We also liked the WhatsApp group*, *so we were messaging each other in-between times…I think it’s good to have that support*.*”*

(Parents)

From legitimizing play, to self-care and ‘seeing it work’, a parent-led approach, relaxed environment, use of vignettes and group discussion facilitated parents’ sense of empowerment. A parent-led approach enabled parents to have ownership over the sessions and to drive the direction of the content:

“*I think probably as I said before*, *group size*, *like mindedness*, *I think just the relaxed atmosphere*. *Totally relaxed*. *And if we were talking amongst ourselves… not amongst ourselves like*, *you know… but if… if the members of the group were having a good discussion*, *the facilitators sort of chipped in but they didn’t try and steer it in any way*, *you know*, *they’d just let that flow*. *And I think that was quite important*, *because we weren’t into this sort of rigid situation*.*”*

(Parent)

### Feasibility

Table 3 presents the three main themes identified in relation to intervention feasibility, including program content and delivery, flexibility of the service, and system fit (supporting quotations are provided in **S3 Table**). Group leaders and service managers perceived a good fit between the program and their service needs but an overall mismatch between service preference to deliver and commissioning arrangements.

**Table 3:** Themes and sub-themes related to feasibility of E-SEE Steps.

1. Nature of program content and delivery
a. Baby versus toddler programs
b. Compatibility with policy initiatives
2. Flexibility of the service
a. E-SEE Steps as a development opportunity
b. Infrastructure–staff roles and levels
c. IY accreditation process
3. System fit

#### Theme 1: Nature of program content and delivery

*Baby versus toddler programs*. From a group leader and service manager perspective, recruitment and retention of parents for IY-I was easier than for IY-T. This is supported by the intervention take-up (84% for IY-I compared to 48% for IY-T) and attrition between acceptance and completion rates for the two programs with a much lower attrition rate for IY-I (IY-I 35%, IY-T 73%):

*I think life is just easier in that first year with a baby*… *That’s when they bring baby with them… And they don’t have lots of other plans, do they? Then with a toddler in tow. Life is just different, isn’t it?*

(Leader)

While services were confident that they would continue to deliver IY-I in the future, there was uncertainty around IY-T due to the practicality of delivery, including difficulty recruiting or engaging parents and ease of implementation. Although most managers and practitioners had expected that IY-T would be easier to implement, issues like crèche provision, difficulty engaging parents and subsequent smaller group sizes, meant that it was experienced as more difficult:

“*I think we’re pretty keen to be running Incredible Babies. I suppose the… the only reservation we currently have is with Incredible Toddlers and just the practical considerations and the financial considerations about providing childcare*, *which…I think it’s become apparent that it’s really necessary to do, but, you know… they showed us the benefits of doing it, but how much of that we will be able to… to manage from a practical point of view*.”

(Service manager)

*Compatibility with policy initiatives*. Although leaders and service managers perceived a good fit between the E-SEE Steps model and their service, several noted a lack of alignment of program content with national guidelines, such as the UNICEF Baby Friendly Initiative (BFI), as problematic going forward. Despite amendments to the IY-B / IY-I book during the external pilot to accommodate BFI standards [12, 36], leaders raised issues in terms of how content aligned with particular guidelines, such as vignette content in IY-I around feeding and stressed that these would need to be fully addressed if they were to deliver the program going forward: *“Yeah*, *this was quite a bone of contention for the health colleagues in that they were very concerned about the bottles in some of the vignettes”* (Leader).

#### Theme 2: Flexibility of the service

*E-SEE as a development opportunity*. Services saw E-SEE Steps as a development opportunity; providing staff with training opportunities in parent support and group facilitation, access to supervision, and active involvement in research:

“*Well, we wouldn’t have been able to access the training. That’s come with this piece of research, training made available to our service*, *so we wouldn’t have had that opportunity to find that funding to provide that and we would definitely not have been able to offer the clinical supervision that we provided in the early stages. So we knew it was something that was right but we didn’t have that research to tap into to make that happen*.”

(*S*ervice manager)

*Infrastructure–staff roles and levels*. Staff level and role were highlighted as issues for long-term feasibility. The IY developer specifies at least two years’ recommended experience for those leading groups. This includes: experience with young children; experience with parenting skills and family interactions; at least one course in child development; and involvement with group activities/awareness of group dynamics. Training in social learning theory is also recommended. Additionally, the developer recommends that at least one of the two leaders conducting a group should have a Master’s degree or higher, or a comparable educational background, with the therapeutic skills needed to assess and manage parents and children with mental health problems. Most group leaders in this study satisfied these criteria, having extensive experience with young children. Although only one group leader held a Master’s degree, many had previous experience in leading group activities/programs. It was suggested by some services that IY delivery would be more feasible in the UK context if it could be delivered by a lower band of staff:

“*So I think all I’m trying to say is, it wouldn’t (be feasible)…we are the 0–5 service. We’re an expensive service*…*And I think that is the… where the issues would lie… But certainly our Band 5 staff would have the flexibility and… to be able to commit to those kinds of programmes more than our Band 6s*.”

(Service manager)

Group leader suitability was also raised by IY mentors who felt that background experience and ability to embrace the collaborative aspects may be more important than band or level of qualification per se:

“…*the usual thing that you see on training is some very strong participants who just seem to get it, and their experience lends themselves to*… *to kind of understanding the programme quickly and being comfortable with role-plays and that kind of thing. And then you get participants who really didn’t, or don’t quite understand what was expected of them, and it’s just not a good fit*.”

(IY mentor)

*IY accreditation process*. In order to ensure capacity to support any future roll out of the E-SEE STEPS model, IY mentors described a need for more group leaders to work towards accreditation. Although many (n = 21, 53.8%) group leaders reported that they were planning on running more IY groups in the future, only seven (17.9%) said that they were working towards becoming accredited. Group leaders cited the process of accreditation as problematic, specifically with regard to financial and logistical support from their organizations. This was further compounded by a lack of capacity in the system, or uncertainty in future capacity of staff in the service to run another three groups to have the video footage evidence of group delivery to submit for accreditation: “*I really*, *really would like to…But again it comes down to budget and if I’m going to be here in six months”* (Leaders).

#### Theme 3: System fit

Service managers felt that the IY programs aligned well with their current parenting offer, often filling gaps in their provision, although both group leaders and service managers agreed that a lack of support and funding at commissioner level would hamper the feasibility of delivery going forward. Managers described a mismatch between the more responsive support they would like to offer their communities and their (highly targeted) commissioning arrangements; they felt it was unlikely they would have the organizational structure to support delivery of E-SEE Steps into the future. This was particularly true for health services, where changes in the remit/roles of practitioners had reduced involvement in parenting programs:

“*The problem is as an organisation we would probably like to do it and support it, but we’re very, very constrained by the commissioning arrangements*… *we can’t agree to do things for which we are not funded. So there is a serious mismatch about that at the moment*.”

(Service manager)

“…*the problem has been in capacity again. The asset we’ve had, which is… really down to one person*, *is that the practitioner who has delivered the program in the [site] area absolutely thinks it’s wonderful… and so I’ve just given her the go ahead… But we are not getting that message from higher up*.”

(Service manager)

Several of the service managers acknowledged that they had underestimated the demand on their service. They also described structural changes to services which were already stretching, or likely to stretch, capacity further, making the time and staff commitment required to deliver E-SEE Steps problematic.

“*I think the difficulty probably was a slight underestimation of the demand on our service, but in conjunction with that, we were going through a huge period of change, probably the biggest the service has ever seen, slightly after agreeing to participate*, *so… and I don’t think at that time, we could have had the insight to know how big an impact those changes would have on our staff and service …It’s all our universal service having to have a big shift to absorb a lot of the work*”

(Service manager)

## Discussion

This study explored the acceptability, feasibility and practical implementation of the E-SEE Steps model, in the context of a randomized controlled trial [see 9]. The trial found no significant impact of the intervention on child or parent outcomes. Despite strong parent satisfaction and practitioner enthusiasm for the program, several barriers to implementation were identified in this process evaluation that might explain the trial’s null effect. Some of these are amenable to change through intervention adaptation and development or by removing research design constraints; others are intrinsic to the complex service system that supports delivery where it is potentially difficult to effect and measure change [see 37]. Our findings support conclusions from a recent similar study of the IY Infant and Toddler programs in the Republic of Ireland [38] with implications for both the program and organizations.

Intervention features that could be revised include: updating program materials to be UK-specific and temporally relevant; ensuring availability of audio/electronic versions of the resources for a wide audience, not just those who struggle with literacy or language; and providing “booster” training or delivery preparation for practitioners to embed the IY collaborative process. As part of the external pilot [see 12, 36], the program developer produced a second version of the IY-B book in line with feedback from sites and standards set by the BFI. However, data from the process evaluation do not support the use of this book as a *stand-alone* resource in its current form; the parents and practitioners interviewed–while only a minority of the trial sample–pointed to the scaffolding that is needed to enable parents to access its content and to the revisions still required to ensure alignment with the current UK early years’ approach. Similar amendments could also be made to the IY-I video and IY-T book and video resources, but it would require additional investment and agreement from the developer to do so.

The trial’s supervision model was challenged by the organizational and system context; among other things, organizational data protection protocols prevented file sharing between co-delivery agencies and the IY Mentors. In addition, despite widespread implementation of IY parenting programs [39], there are few certified UK IY Mentors and resource constraints meant that travel for face-to-face supervision was not feasible during the trial. This has implications for future implementation; significant investment is needed in UK systems to upskill the workforce to fulfil this critical supervisory function, preferably at a local level. IY Inc. USA provides a structured system of accreditation to move from local group leader to mentor and trainer but the process takes investment and hours of training, delivery and supervision. This makes capacity building a difficult prospect for overstretched public service systems, which experience high staff turnover in the context of limited resources for professional development [6].

Features of the children’s services’ system that require consideration if PU models are to be supported, include the alignment between national mandates, locally commissioned service offers and the capacity of providers to deliver intensive programs like IY. At the start of the pilot trial in 2015, responsibility for Health Visiting passed to the Local Authorities in England, resulting in lengthy service transformation programs. These changes directly impacted health visitors’ roles, largely reducing their involvement in the delivery of parenting support and affecting their capacity to deliver E-SEE Steps. In addition, our findings suggest that multi-agency collaboration is a necessary but not sufficient condition to deliver a model like E-SEE Steps, which assumes a shared culture and approach. Sites with existing multi-agency partnerships were significantly quicker in setting up and implementing the model but this did not guarantee successful recruitment or retention of parents to the IY groups.

Indeed, take-up of the targeted group-based programs was lower than expected, particularly for IY-T, and many eligible parents were “lost” before the first session of the groups, i.e., they did not engage with the offer at all. Our findings suggest this is most likely due to parents returning to work and the lack of flexibility in the timing of group delivery (e.g., evenings or weekends), but also because familiarity and trust had not been sufficiently established with those parents previously unknown to the services. The evidence suggests that greater investment in building rapport and motivation to change in parents, prior to groups starting, could help to boost access and retention [40, 41]. This engagement with services is also likely to be more effective if it begins antenatally when expectant parents may have greater capacity to consider health education. Lack of staff capacity presented challenges to undertaking this engagement work in trial sites, and may have particularly impacted parents who struggled with low mood and anxiety who needed more support/engagement to facilitate group participation. Other intervention wrap-around support such as childcare and transport assistance were often difficult for services to provide because of limited financial resources and are unlikely to be commonplace support offers in real world delivery.

This research is one of the few process evaluations of a parenting intervention, delivered via three steps over the course of the child’s early life, focused on children in the early years and has included the views of four key stakeholders, including families who were eligible but who did not engage with the targeted steps of the intervention. While the study included a large sample in qualitative terms, it is limited by the fact that the group of parents who participated are unlikely to represent all parents in the larger trial. In addition, the study did not explore the experience of families who received only the universal component of the model; conclusions about IY-B as a universal resource have been drawn from those parents who were also eligible for one of the targeted programs. Finally, while this study has surfaced a number of critical organizational and service-level barriers to delivery, it was not possible within the resources of the trial to examine in detail the influence of wider system-level context on the intervention, such as the receptivity of sites to a PU model (though site selection involved a site-completed readiness proforma) or the presence/absence of local policies or strategies that may have facilitated its adoption or implementation.

## Conclusion

Marmot and colleagues highlighted the need for high quality parenting programs to meet needs across the social gradient, a goal which they claim has seen limited progress in the 10 years since their first report [6]. However, the success of PU models, like E-SEE Steps, is contingent on both the faithful delivery of the intervention programs and the supportive, flexible and resilient organizational systems that host and deliver them. In this instance, the model assumed that existing home visiting and children’s services’ support were sufficient to provide the opportunities for family assessments and to convey information about community groups relevant to parenting needs. However, our evaluation findings point to real difficulties for practitioners and service organizations in ensuring the resources and capacity to identify eligible parents, properly engage and retain them, and to prepare sufficiently to deliver programs with fidelity, with appropriate supervision and oversight. Taken together, these findings provide a reasonable explanation for the lack of positive impact demonstrated in the effectiveness study and a need for further, more focused research is indicated.

## Supporting information

S1 Fig E-SEE Steps model. (DOCX)

S1 File Focus group/interview topic guides with parents (A), IY group leaders (B), service managers (C), and IY mentors (D).
(DOCX)

S1 Table Summary of incredible years content. (DOCX)

S2 Table CSRI reported childcare and parenting class utilization in the E-SEE trial arm (A) and services as usual (SAU) trial arm (B).
(DOCX)

S3 Table Summary of themes of barriers and facilitators to delivering E-SEE Steps (A), and themes for acceptability and feasibility of E-SEE Steps (B).
(DOCX)

S4 Table Practitioner characteristics (A) and practitioner confidence in setting up and running a parenting group (B).
(DOCX)
