# Supplementary material for: Barriers and facilitators in the delivery of a proportionate universal parenting program model (E-SEE Steps) in community family services
Source: PLoS One. 2022 Jun 13;17(6):e0265946. doi: 10.1371/journal.pone.0265946 (PMC9191704; doi:10.1371/journal.pone.0265946)
Supplement: S1 File — Focus group/interview topic guides with parents (A), IY group leaders (B), service managers (C), and IY mentors (D). (DOCX) [file pone.0265946.s002.docx]

**S1A. Focus group/interview topic guide with parents**

**Topic Guide 1:**

Focus groups with parents attending 1 or more sessions of the Incredible Years Baby and/or Toddler programme (1-2 weeks after completion of final outcome data when child is ~20 months)

Time allowed: 2 hours including welcome, introductions and refreshments break.

Group no larger than 10 people

Venue –

**Aims of the study**

This study aims to understand whether the Incredible Years parenting programmes (baby and toddler) are acceptable and practical interventions to use with carers, of young children under two years of age. It also aims to explore the aids and barriers to successful delivery of the programme/s.

**Introduction**

The trial is being led by the University of York. The process evaluation is being led by the University of Exeter. This group discussion focuses on how parents who attended at least one of the parenting group sessions experienced the programme, including recruitment and retention strategies.

Focus group facilitators explain:

It is a discussion not a question and answer session

Tape/audio-recording

Confidentiality

**Let’s start by introducing ourselves.** *Can you each say your name and a little bit about yourself, e.g. where you are from, which group/s you attended, number and ages of children, a hobby.*

**We are keen to understand how well the processes to identify and recruit people to the E-SEE study worked.** *How did you experience the process of being recruited to the study and being invited to take part in a parenting group?*

Prompts:

- Who told you about the study?
- Were you given enough information about the programme before the health visitor passed on your details to the research team?
- Did you get the information you needed from the research team about what was involved in taking part in the study?
- Why did you ultimately decide to take part?
- Is there any additional information or contact you would have liked/needed to aid your decision about whether to take part?

**You have all been selected for this discussion because you have attended sessions from either/both the Incredible Years Baby and Toddler programmes.**

*How useful did you find the programme/s you attended, and how (if at all) has it impacted on your daily life?*

Prompts:

- Did you learn anything new?
- Did you discover/try out new strategies for interacting with your baby/toddler?
- What did you like most/least about the programme?
- Did you struggle with low mood or parenting before going on the programme? If so, did that change?
- For those who attended both programmes, did you find one more useful than the other?

**We would like to understand the things that enable or hinder people’s ability to attend the groups.**

1. *Were there circumstances or issues that meant you could not attend sessions when you would have liked to?*
2. *Were there things offered/done that particularly encouraged you to attend?*

Prompts:

- Did the parenting group you attended have a crèche? Did you use it? If it didn’t have one, did you need one?
- Could you access transport to/from the venue easily? Did you use public transport?
- Were the group meetings at convenient times for you?
- Were you concerned about group leaders or individuals within the group, e.g. someone you disliked/felt uncomfortable around, or gender of group members?
- Did low mood/emotions prevent you from attending any sessions?
- Did you make friends/network with anyone in the group that particularly encouraged you to attend?
- Did the group leader contact you before or in between sessions – did that encourage you to attend?

**Each programme has an accompanying handbook, and the Baby book was given out to all parents.** *How useful (if at all) did you find the books provided as part of the programme?*

Prompts:

- Did you use the book at home, e.g. to try out the exercises or complete the homework?
- Was the language used clear and understandable? Were there words used you did not like/understand?
- Was the book presented in an engaging way, e.g. colour, pictures, humour?
- If you had access to an audio version, how useful was that?

***Finally, how do you think the things you have learned from the group or the friends/networks you have formed might benefit you in the future?***

**S1B. Focus group topic guide with IY group leaders**

**Topic Guide 2:**

Focus groups with facilitators that have delivered either/both the Incredible Years Baby and/or Toddler programme (after programme delivery)

Time allowed: 1 hour including welcome and introductions.

Group no larger than 10 people

**Aims of the study**

This study aims to understand whether the Incredible Years parenting programmes (baby and toddler) are acceptable and feasible interventions to use with carers, of young children under two years of age, who have low mood or depression. It also aims to explore the aids and barriers to successful delivery of the programme/s.

**Introduction**

The trial is being led by the University of York. The process evaluation is being led by the University of Exeter. This group discussion focuses on how facilitators who delivered at least one parenting group experienced programme set-up and delivery, including training and supervision, and organisational infrastructure/support.

Focus group facilitators explain:

It is a discussion not a question and answer session

Tape/audio-recording

Confidentiality

**Let’s start by introducing ourselves.** *Can you each say your name and a little bit about yourself, e.g. your job/role.*

**You have all been selected for this discussion because you have delivered/co-delivered either or both the Incredible Years Baby and Toddler programmes.**

1. *How instructive did you find the training provided to qualify you to deliver the programme? [2 day training for Baby, 3 day training for Toddler programme]*
2. *How useful was the supervision provided?*

Prompts:

- Had you delivered other IY programmes before the E-SEE study?
- Was the official training too long, too short, or just right?
  - Explore usefulness of refresher/top-up/workshop sessions.
- Was there adequate time for preparation and reflection?
- Were the trainers/supervisors engaging and supportive?
- Did the size of the group trained/supervised make a difference to your experience of the training?
- Did you attend the supervision offered? Did you change anything about your delivery of the programme/s as a result of the supervision sessions?
- Was the format of the supervision acceptable, i.e. phone rather than face to face?

**Each programme has an accompanying handbook, and the Baby book was given out to all parents.** *How useful do you think the programme books were for parents in your groups? Did you find that parents used/referred to the books?*

Prompts:

- Did you direct parents to the book, during the session or for homework purposes? Did parents bring the books to the sessions?
- Were the books suitable for different populations/audiences, e.g. low literacy or ethnic/language minorities?
- Did any difficulties arise with respect to the US English translations?
- Did any difficulties arise with the contradictions between the IY Book and BFI guidelines?

**We would like to understand the things that enable or hinder people’s ability to accept and attend the groups?**

*Do you think there were particular reasons why parents did/did not accept the offer or attend the group sessions you delivered?*

Prompts:

- Were any of the individuals within the group disruptive or difficult to control?
- Did low mood/emotions prevent individuals from participating in sessions?
- Were any activities/components particularly well received by parents?
- Did parents in general actively engage in the sessions?
- Did you contact parents before or in between sessions?

**Do you think the research process helped or hindered how you ran the sessions?** Is there anything you would have liked, in the way of support or input or the format thereof, from the research team that you didn’t get?

**How do you think your organisation/s either supported or hindered your ability to deliver the programmes successfully?**

Prompts:

- Were you given an option to train/deliver or not?
- Were you given enough time for preparation and session set-up?
- Was there institutional support for supervision?
- Were you encouraged to work across disciplines or outside of your own organisation, e.g. between health and children’s services?
- Is there anything you would have liked, in the way of support, from your organisation to help you run the programme that you didn’t get?

*Finally, how do you think being trained in the Incredible Years and/or delivering it has changed your professional practice? Do you think you will continue to deliver the Incredible Years programme, and become accredited? Where could funding/support for this come from?*

**S1C. Interview topic guide with service managers**

**Topic Guide 3:**

Interviews with service managers who have co-ordinated or overseen the E-SEE STEPS set-up and delivery

Time allowed: 1 hour

**Aims of the study**

This study aims to understand whether the E-SEE STEPS model - comprising a universal level of Incredible Babies book provision, a targeted Incredible Years baby parenting programme, and a targeted Incredible Years Toddler parenting programme, - is an acceptable and feasible intervention to use with carers, of young children under two years of age, who have low mood or depression. It also aims to explore the aids and barriers to successful delivery of the model.

**Introduction**

The trial is being led by the University of York. The process evaluation is being led by University of Exeter. This interview discussion focuses on understanding the service context within which the E-SEE STEPS model was delivered, including the organisational and system facilitators and barriers, as well as service managers’ experience of the trial processes.

Explain:

Tape/audio-recording

Confidentiality/anonymity

Who does this view represent?

1. **Can you explain your job/role in your organisation, and your involvement in the delivery of the E-SEE interventions?**

Prompt:

- Have you supervised staff delivering the interventions, or been responsible for allocating resources, etc?

Organisational context

1. **Your organisation took the decision to partner in the E-SEE study and to deliver the E-SEE STEPS model. Could you tell me about that decision-making process, who was involved, and the factors considered?**

Prompts:

- Was cost/resource a factor?
- How did any previous experience of the Incredible Years influence your decision to take part?
- Was staff training/skills level a factor?
- Do you think your service would have chosen to implement Incredible Years if it was not part of the E-SEE research study?

1. **Have there been any changes during the pilot period to the work/focus, structure or people within your organisation that might impact on that decision-making process, i.e. to be part of a study or implement E-SEE STEPS?**

Prompts:

- Key staff changes (e.g. CEO, HoS, LA leadership)?
- Corporate strategy, target population, nature or scope of the roles?
- Targets or performance indicators?
- Role structures/hierarchy?

1. **How would you describe the fit between the E-SEE model (and/or IY programmes) and your organisation’s parenting or mental health strategies?**

Prompts:

- Does your service have a parenting strategy?
- What is the strategy regards post-natal depression?
- Have these strategies changed in the last 2 years?
- Do you work using a universal-proportionate approach generally, or has E-SEE STEPS been the first UP model you have implemented?

Service/implementation context

1. **Have there been any particular challenges or difficulties in implementing the E-SEE IY programmes in your service? *We are interested in both organisational/system challenges as well as team/individual difficulties with the intervention.***

Prompts:

- Does the programme fit with the organisational culture?
- How have guidelines (e.g. BFI or DoH) either constrained or promoted the programmes?
- Has cost/required resources been an issue? Provision of creche, transport, etc
- Do staff like/dislike the programmes?
- Has staff capacity been an issue?

1. **(If not covered above) How could the recruitment of parents to E-SEE be improved?**
2. **Have you had to make any accommodations or adaptations to the way you run your service in order to deliver E-SEE STEPS/IY?**
3. **E-SEE STEPS has been piloted as a co-delivery model between HV and CS. Are there partners it would have been useful to involve or co-deliver with? Has this been a challenge for your service?**

Prompts:

- What arrangements were already in place for cross-disciplinary working in your area?
- Has anything changed in the relationship with other service partners since the start of E-SEE?
- Are the relationships sustainable?
- What supports positive working relationships? What are the threats to positive working relationships?

1. **What communication strategies have you employed to inform and/or motivate staff responsible for the recruitment to or delivery of E-SEE STEPS?**

The research process

1. **E-SEE Steps is part of a research trial and has therefore likely involved processes that may be different to your usual working practices. *What is your understanding of the purpose of the research? Did this change from when you signed up to the study?***

Prompts:

- Did you have access to enough information about the research from the outset?
- Was the research team able to answer any questions that you had about the trial?

1. **Could you tell me a bit about your experience of being involved in the research? Did the process create any particular challenges for you individually, your team or the wider service?**

Prompts:

- Dealings with the clinical trials unit (Sheffield Uni.)
- Excess treatment costs (for Health organisations)
- Site set up, e.g. service design meetings, identification briefings, service level agreements, etc.

1. **Is there anything else that the research team could have done to make it easier for you to participate? Could any aspects of the trial be changed to make it a better fit for your organisation?**

Prompts:

- E.g. additional documents/information about the E-SEE model or study?
- Communication frequency/format with the research team?
- Participant identification/recruitment procedures and screening methods?

1. **Have you / your service made use of the programme fidelity information on attendance or adherence?**

Anticipated Effects/Future

1. **Has the E-SEE STEPS model changed services, relationships and/or professional practice in your area?**
2. **Do you think your service will continue to deliver the E-SEE STEPS model if there is funding/support for it?**

**S1D. Interview topic guide with IY mentors**

**Topic Guide 4:**

Interviews with IY mentors who have overseen the training and supervision of the E-SEE group delivery.

Time allowed: 30 minutes

**Introduction**

The trial is being led by the University of York. The process evaluation is being led by University of Exeter. This discussion aims to focus on understanding the E-SEE training delivery and supervision context and challenges faced by mentors and mentees (group facilitators) during the course of the trial.

**Explain and refer to Info. sheet:**

Tape/audio-recording – for note taking purposes. Will be deleted once transcribed.

Confidentiality/anonymity – we will want to use quotes from this interview for our report/papers. If it may identify you, we will seek your permission to do so.

Who does this view represent?

1. **Can you BRIEFLY explain your job/role, and your involvement in the E-SEE trial?**

Prompt:

- How long have you been an IY mentor?
- What ESEE sites have you provided supervision for and for which IY programmes in each site (IYI/IYT)?
- Is this over and above being an IY mentor/trainer? *Sustainability issue?*

Training delivery

1. **Could you reflect on the delivery of the IY training?**

Prompts:

- How well did the training sessions go in each site you delivered it?
- Were there differences across sites?
- If you delivered both IYT and IYI training can you reflect on any differences you experienced in delivering both trainings?
- What were the challenges? Where do they stem from (IY content/trainee facilitator behaviour or qualifications/delivery context)?
- Any thoughts on how these challenges could be addressed?
- What went well during the training?
- How did you feel the training was received by trainee facilitators? i.e. were they engaged/keen to participate in role play etc?

1. **Are there any changes or recommendations you would make to improve the delivery of the IY training going forward?**

Prompts:

- - Adequate support/resource/appropriate space?
  - Level and type of experience of those training?
  - Time for training?

Supervision and support

1. **What is your perception of how valuable the supervision was for the facilitators?**

Prompts:

- - Did you feel the sessions were productive?
  - Were facilitators motivated to engage in supervision?
  - Did they bring real problems to the supervision session?

1. **Can you reflect on the format of supervision, what worked well/not so well?**

Prompts:

- - Experience engaging in supervision over phone/skype with no video content to feedback on, what affect did this have?
  - Number of supervision sessions
  - Facilitator engagement with this format of supervision

1. **To what extent do you feel your supervision actually changed practice?**

Prompts:

- - Did you have to help facilitators think through any particular adaptations/ accommodations to delivery? E.g. for small groups or dominant parents.
  - Were facilitators keen to make adaptations/apply feedback from supervision sessions?

1. **Were there any components/strategies of either IYI or IYT, or both, that came up consistently as difficult to deliver? If so, what are they and why do you think it was problematic?**

Prompts:

- - E.g. Role play/ Compliance with sessions – fidelity in terms of content and number of sessions / Small group sizes – management and how this changes the group dynamic/retention
  - Did you feel like the foundation qualifications/service discipline of the facilitators made a difference to their ability to deliver?
  - How could we overcome these challenges?

1. **What do you foresee as the challenges for scale up/ roll out of the E-SEE model?**

Prompts:

- - Videos/accreditation/retention and group size?
  - What could be done to support the roll out in order to overcome these challenges?
