# Supplementary material for: Barriers and facilitators in the delivery of a proportionate universal parenting program model (E-SEE Steps) in community family services
Source: PLoS One. 2022 Jun 13;17(6):e0265946. doi: 10.1371/journal.pone.0265946 (PMC9191704; doi:10.1371/journal.pone.0265946)
Supplement: S1 Table — (DOCX) [file pone.0265946.s003.docx]

**S1 Table.** **Summary of Incredible Years^®^ Content**
