# Supplementary material for: Barriers and facilitators in the delivery of a proportionate universal parenting program model (E-SEE Steps) in community family services
Source: PLoS One. 2022 Jun 13;17(6):e0265946. doi: 10.1371/journal.pone.0265946 (PMC9191704; doi:10.1371/journal.pone.0265946)
Supplement: S2 Table — CSRI reported childcare and parenting class utilization in the E-SEE trial arm (A) and services as usual (SAU) trial arm (B). (DOCX) [file pone.0265946.s004.docx]

**S2A Table. CSRI reported childcare and parenting class utilization in the E-SEE trial arm**

|  |  |  | **E-SEE Steps** | | | | | | | | | | | | | | | | | | | | | | | | |
| --- | --- | --- | --- | --- | --- | --- | --- | --- | --- | --- | --- | --- | --- | --- | --- | --- | --- | --- | --- | --- | --- | --- | --- | --- | --- | --- | --- |
|  |  |  | **Baseline** | | | | | **Month 2** | | | | | **Month9** | | | | | **Month 18** | | | | | **Total** | | | | |
|  |  |  | **N** | **Mean** | **SD** | **Min** | **Max** | **N** | **Mean** | **SD** | **Min** | **Max** | **N** | **Mean** | **SD** | **Min** | **Max** | **N** | **Mean** | **SD** | **Min** | **Max** | **N** | **Mean** | **SD** | **Min** | **Max** |
| ***Childcare*** | |  |  |  |  |  |  |  |  |  |  |  |  |  |  |  |  |  |  |  |  |  |  |  |  |  |  |
|  | Playgroup (hours) | | 32 | 17.75 | 13.59 | 8 | 56 | 46 | 73.20 | 57.49 | 1 | 280 | 69 | 21.97 | 25.69 | 1 | 200 | 54 | 22.20 | 16.58 | 8 | 80 | 109 | 52.61 | 60.25 | 1 | 368 |
|  | Childminder (hours) | | 0 | - | - | - | - | 2 | 56.00 | 39.60 | 28 | 84 | 32 | 119.00 | 93.58 | 8 | 320 | 42 | 152.38 | 78.58 | 24 | 320 | 46 | 213.74 | 167.53 | 8 | 560 |
|  | Friend or family member (hours) | | 19 | 40.84 | 42.57 | 8 | 160 | 15 | 169.87 | 178.49 | 28 | 672 | 74 | 113.19 | 78.96 | 8 | 320 | 120 | 123.60 | 100.02 | 8 | 768 | 137 | 178.95 | 152.27 | 8 | 768 |
|  | Day nursery (hours) | | 3 | 53.33 | 57.87 | 16 | 120 | 5 | 112.00 | 79.20 | 28 | 196 | 49 | 152.33 | 92.12 | 8 | 400 | 101 | 171.25 | 86.32 | 32 | 400 | 103 | 242.02 | 159.94 | 28 | 800 |
| ***Parenting courses*** | | |  |  |  |  |  |  |  |  |  |  |  |  |  |  |  |  |  |  |  |  |  |  |  |  |  |
|  | Triple P |  | 1 | 1.00 |  | 1 | 1 | 0 | - | - | - | - | 0 | - | - | - | - | 0 | - | - | - | - | 1 | 1.00 | - | 1 | 1 |
|  | Family Nurse Partnership Groups | | 0 | - | - | - | - | 0 | - | - | - | - | 0 | - | - | - | - | 0 | - | - | - | - | 0 | - | - | - | - |
|  | Family Nurse Partnership 1:1 | | 0 | - | - | - | - | 0 | - | - | - | - | 0 | - | - | - | - | 0 | - | - | - | - | 0 | - | - | - | - |
|  | Solihull Parenting Programme | | 1 | 1.00 | - | 1 | 1 | 0 | - | - | - | - | 0 | - | - | - | - | 0 | - | - | - | - | 1 | 1.00 |  | 1 | 1 |
|  | Home Start | | 1 | 1.00 | - | 1 | 1 | 2 | 1.00 | 0.00 | 1 | 1 | 8 | 1.00 | 0.00 | 1 | 1 | 2 | 1.00 | 0.00 | 1 | 1 | 13 | 1.00 | 0.00 | 1 | 1 |
|  | Family Links | | 0 | - | - | - | - | 0 | - | - | - | - | 0 | - | - | - | - | 0 | - | - | - | - | 0 | - | - | - | - |
|  | Northamptonshire baby programme | | 0 | - | - | - | - | 0 | - | - | - | - | 0 | - | - | - | - | 0 | - | - | - | - | 0 | - | - | - | - |
|  | Happy |  | 0 | - | - | - | - | 0 | - | - | - | - | 0 | - | - | - | - | 0 | - | - | - | - | 0 | - | - | - | - |
|  | Babysteps | | 0 | - | - | - | - | 0 | - | - | - | - | 0 | - | - | - | - | 0 | - | - | - | - | 0 | - | - | - | - |

**S2B Table. CSRI reported childcare and parenting class utilization in the services as usual (SAU) trial arm**

|  |  | | **SAU** | | | | | | | | | | | | | | | | | | | | | | | | | | | | | | | | | | | | | | | | | | | | | | | | |
| --- | --- | --- | --- | --- | --- | --- | --- | --- | --- | --- | --- | --- | --- | --- | --- | --- | --- | --- | --- | --- | --- | --- | --- | --- | --- | --- | --- | --- | --- | --- | --- | --- | --- | --- | --- | --- | --- | --- | --- | --- | --- | --- | --- | --- | --- | --- | --- | --- | --- | --- | --- |
|  |  | | **Baseline** | | | | | | | | | | **Month 2** | | | | | | | | | | **Month9** | | | | | | | | | | **Month 18** | | | | | | | | | | **Total** | | | | | | | | |
|  |  | | N | | Mean | | SD | | Min | | Max | | N | | Mean | | SD | | Min | | Max | | N | | Mean | | SD | | Min | | Max | | N | | Mean | | SD | | Min | | Max | | N | | Mean | | SD | | Min | | Max |
| ***Childcare*** | |  | |  | |  | |  | |  | |  | |  | |  | |  | |  | |  | |  | |  | |  | |  | |  | |  | |  | |  | |  | |  | |  | |  | |  | |  |  |
| Playgroup (hours) | | | 3 | | 21.33 | | 16.65 | | 8 | | 40 | | 8 | | 56.00 | | 25.92 | | 28 | | 112 | | 11 | | 26.91 | | 32.32 | | 6 | | 120 | | 7 | | 19.43 | | 16.56 | | 8 | | 56 | | 18 | | 48.89 | | 48.10 | | 8 | | 200 |
| Childminder (hours) | | | 0 | | - | | - | | - | | - | | 0 | | - | | - | | - | | - | | 5 | | 174.40 | | 123.47 | | 16 | | 360 | | 7 | | 305.14 | | 62.38 | | 224 | | 400 | | 7 | | 429.71 | | 143.24 | | 288 | | 720 |
| Friend or family member (hours) | | | 5 | | 56.00 | | 58.79 | | 8 | | 128 | | 7 | | 400.00 | | 385.52 | | 28 | | 1120 | | 11 | | 93.09 | | 61.69 | | 16 | | 200 | | 19 | | 101.47 | | 76.78 | | 8 | | 320 | | 25 | | 218.88 | | 303.61 | | 16 | | 1192 |
| Day nursery (hours) | | | 0 | | - | | - | | - | | - | | 2 | | 56.00 | | 39.60 | | 28 | | 84 | | 10 | | 199.20 | | 105.62 | | 64 | | 360 | | 21 | | 165.33 | | 94.40 | | 32 | | 360 | | 21 | | 257.90 | | 212.34 | | 32 | | 720 |
| ***Parenting courses*** | | | | |  | | |  | |  | |  | |  | |  | |  | |  | |  | |  | |  | |  | |  | |  | |  | |  | |  | |  | |  | |  | |  | |  | |  |  |
| Triple P |  | | 0 | | - | | - | | - | | - | | 1 | | 1.00 | | - | | 1 | | 1 | | 1 | | 1.00 | | - | | 1 | | 1 | | 1 | | 1.00 | | - | | 1 | | 1 | | 1 | | 3.00 | | - | | 3 | | 3 |
| Family Nurse Partnership Groups | | | 0 | | - | | - | | - | | - | | 0 | | - | | - | | - | | - | | 0 | | - | | - | | - | | - | | 0 | | - | | - | | - | | - | | 0 | | - | | - | | - | | - |
| Family Nurse Partnership 1:1 | | | 0 | | - | | - | | - | | - | | 0 | | - | | - | | - | | - | | 0 | | - | | - | | - | | - | | 0 | | - | | - | | - | | - | | 0 | | - | | - | | - | | - |
| Solihull Parenting Programme | | | 0 | | - | | - | | - | | - | | 0 | | - | | - | | - | | - | | 0 | | - | | - | | - | | - | | 0 | | - | | - | | - | | - | | 0 | | - | | - | | - | | - |
| Home Start | | | 0 | | - | | - | | - | | - | | 0 | | - | | - | | - | | - | | 1 | | 1.00 | | - | | 1 | | 1 | | 0 | | - | | - | | - | | - | | 1 | | 1.00 | | - | | 1 | | 1 |
| Family Links | | | 0 | | - | | - | | - | | - | | 0 | | - | | - | | - | | - | | 0 | | - | | - | | - | | - | | 0 | | - | | - | | - | | - | | 0 | | - | | - | | - | | - |
| Northamptonshire baby programme | | | 0 | | - | | - | | - | | - | | 0 | | - | | - | | - | | - | | 0 | | - | | - | | - | | - | | 0 | | - | | - | | - | | - | | 0 | | - | | - | | - | | - |
| Happy |  | | 0 | | - | | - | | - | | - | | 0 | | - | | - | | - | | - | | 0 | | - | | - | | - | | - | | 0 | | - | | - | | - | | - | | 0 | | - | | - | | - | | - |
| Babysteps | | | 0 | | - | | - | | - | | - | | 0 | | - | | - | | - | | - | | 0 | | - | | - | | - | | - | | 0 | | - | | - | | - | | - | | 0 | | - | | - | | - | | - |
