# Supplementary material for: Barriers and facilitators in the delivery of a proportionate universal parenting program model (E-SEE Steps) in community family services
Source: PLoS One. 2022 Jun 13;17(6):e0265946. doi: 10.1371/journal.pone.0265946 (PMC9191704; doi:10.1371/journal.pone.0265946)
Supplement: S3 Table — Summary of themes of barriers and facilitators to delivering E-SEE Steps (A), and themes for acceptability and feasibility of E-SEE Steps (B). (DOCX) [file pone.0265946.s005.docx]

**S3A Table. Summary of themes of barriers and facilitators to delivering E-SEE Steps**

| Theme | Sub-theme | Supporting quotes |
| --- | --- | --- |
| Individual level factors | Connection and relevance | *“I seemed to speak to them a lot before we started… Backwards and forwards so I felt that we all really knew each other quite well, really…But that was quite nice because I think they needed that support to…Come join the group”* (Leaders)  *“…when it comes onto this stage of development where the babies were at, it was like… you could see that they actually were more interested and taking on board what… and because they could relate it to their own children, “Oh, mine is doing that,” so it is definitely… it engages them more in that….And I think that is the art of IY actually…it is that real personalisation. That makes sense doesn’t it? If it is pertinent to you, you are going to be engaged in it, you are going to be interested. You are going to feel like you are getting a lot out of it whereas if it is not then not necessarily.”* (Leaders) |
| Organizational level factors | Engagement processes  The IY program  Supervision and mentorship | *“And things change from that initial conversation, that point where they were recruited at the beginning, maybe that mood has improved, maybe they weren't struggling so much with baby”* (Leaders)  *“We’re working on quick timescales so if we say a family is identified for a group we would be hoping that at the start of the next group we’ll be forecasting for planning stuff when one is looking towards the middle, we’re already starting our next group, that then families will be accessing straightaway so they haven’t lost that momentum. They know what it is, they’re not waiting a long time.”* (Service manager)  *“It’s really about maintaining that contact and identifying when is the right time because for a lot of parents you don’t walk past your front door for quite some time when you’ve had a newborn baby. You’re getting your routine in place…”* (Service manager)  *“From our point of view it was… it needed to be the early identification from the visits and from the midwives and from the health visitors trying to sell the… the project really…And we didn’t feel that was happening as much as it could be doing. If they were the ones with the information… we… we don’t have knowledge of which parents and parents to be that are out there, so we need to rely on our partners to be able to do that. So, I… I would just encourage the buy-in from other partners really.”* (Service manager)  *“The kind of thing that came up when I was contacting parents for toddler group was that we didn't have the crèche and we weren't sure if we were going to have a crèche…So that was kind of like yeah, I can come if that's…there but if not, definitely not.”* (Leaders)  *“A couple of them were resistant, very resistant, to role play anyway. They were resistant in the training and they, that moved on and although we spent, I spent more time than anything else I think on trying to problem solve that with them.”* (IY Mentor)  *“I think a common theme would be getting them to not give the parents the direct answers. So where a parent would say… “Well what if I do this? Or should I do this?” And in their normal roles it would be very appropriate for them obviously to give a direct response. So it was encouraging them to be more collaborative… And I think the more they did that, I think they saw that the parents were much more engaged.”* (IY Mentor)  *“And the particular challenge was for us, with… the babies that we had, and I can’t remember why this happened now, but they were four months old when it started, not [eight weeks], and that was quite a challenge, trying to tailor… still deliver the program how it should have been delivered.”* (Leaders)  *“There were elements of it that I thought maybe if there'd been a little bit more around the kind of practical bits of it and more around the actual content of it that would have been beneficial for once we actually ran it.”* (Leaders)  *“*…*very good at actually encouraging you, but also honing in on the needs of the people you are working with, and getting that bit where… because it can be quite prescriptive. It is that bit about getting it to be individualised and personalised to the people that were in the group. They [IY Mentors] were very good at that, and all the wealth of experience comes out, doesn’t it, when you are talking to them.”* (Leaders)  *“[Name of IY mentor] was on the phone, and then you would watch her live, so that experience was really good, and I found that was better than doing a Skype supervision, which we then moved onto. Because the Skype supervision, you were relying on phone connections, everyone else's internet, it didn’t work, and you couldn’t unpick your videos, and I think that's what you need for the incredible years.”* (Leaders) |
| System/service level factors | Support for implementation | *“…not just the time it took to deliver the group, and the time that we blocked out for that, it was the supervision and the… and the prepping of the next group and things like that…that was sort of underestimated, I think, the time, just the time that it…You need to dedicate, and actually people need to be able to be prepared to deliver that group, and deliver it, you know, how it should be delivered.”* (Service manager)  *“On my manager's side she's kind of just given me free rein to, you know, manage my time around it and I didn't feel pressure from her, I felt that actually we could just do it and manage it ourselves, which felt pretty good. But she sort of said, you know, we need to do this…”* (Leaders) |

**S3B Table. Summary of themes for acceptability and feasibility of E-SEE Steps**

| Theme | Sub-themes | Supporting quotes |
| --- | --- | --- |
| **Acceptability** | | |
| Nature of the intervention offer | Receiving and understanding the offer  Familiarity  Creche and childcare  Co-parent attendance | *“I didn’t have any [information]. All mine was, was when the lady [a trial researcher] come to my house she kept saying I don’t want to hear if you’re going or not and I just got invited to it but I didn’t have a clue what it was. Until I actually got there…”* (Parent)  *“Yeah, I think it was the health visitor. But I don’t think she knew much about the study…But she just mentioned it and asked me if I’d be happy to…Take part in the study”.* (Parent)  *“Yeah, I think that would have helped, knowing at least one other person that was going. Obviously you don’t know anyone. That probably would have been more encouraging.”* (Parent)  *“A more local group would probably have been easier…It was going to be two groups, wasn’t it, that they didn’t get enough to do the [name] one, which is why you came here…If it was further away I don’t know if I would have made it. Being local is probably imperative.” (Parent)*  *“I’m not on maternity leave anymore, it meant giving up quite a chunk of time with my kids on the only day I have off with them… And I didn’t really understand why they had to be in a separate room*.*”* (Parent)  *“…a few of them did say, yeah, we're coming back and then just before the coffee morning a couple of them basically turned around and said no, we just can't do it…We haven't got the time, it's our only time off with the little ones, we just want to spend time with them.”* (Leaders)  *“I don’t think it was something that was made clear but, even if it was, he works so he wouldn’t have been able. I’m self-employed but on maternity leave at the time, my partner, he wouldn’t have been able to get time off. He’s an engineer so he’s pretty much always on call.”* (Parent)  *“Better if you could bring somebody with you…So not… I know you're a single mum but if you could bring like your mum with you…”* (Parent) |
| Demands of attendance and programme processes |  | *“The other thing I didn’t like, the role play, that was so cringe…One of us was the child and the other was the mum”*  *“Yeah like throwing a tantrum or doing something they shouldn’t…It felt awkward cos you can’t really say ‘oh no don’t do that!’ It didn’t feel like comfortable yelling at someone”*  *“We kept getting the leaders to do it cos… They wanted us to practice giving praise and um we wouldn’t do it so we had the leaders and one of them was putting toys away and the other one was praising them and you could just see she’d gone bright red”* (Parents)  *“I suppose you've got that additional barrier with some of the participants that you are targeting. That definitely was a case in our last toddler, there was someone whose mental health deteriorated so they couldn’t continue… mood and baby IY is usually the barrier as well, just the fact that they have got the baby, it is really hard to get out to a group, just in general, I think that's an additional barrier isn't it?”* (Leaders)  *“…the first time when they were babies I couldn't go because my anxiety manifests as agoraphobia, and I couldn't get… I just couldn't get out the house.”* (Parent)  *“Yeah, and I didn’t go only because I was suffering quite bad with anxiety at the time and I didn’t feel comfortable going without my partner.”* (Parent) |
| Utility of IY Baby books |  | *“I looked at it, yeah…Some bits I kind of like flicked and then certain bits. But not, in all honesty, once the group started, I didn’t really go back to the book.”* (Parent)  *“As it’s anonymous, I’m going to admit that I didn’t read the book.”* (Parent)  *“A lot of it’s intellectual. So it’s really hard to understand…So it’s like, what’s that word? Google it.”* (Parent)  *“Thing is I think it's quite a difficult read and I…did as I was reading it think I bet there's some people that won't bother reading this…But obviously if it coincides with the course it's a different matter but it's… if you're just handed the book…I think it could be quite intimidating…”* (Parent) |
| Nature of a parenting group |  | *Well, because it was my first, I have no friends with babies. In my office no-one had kids. They’re all my age so for me it was also like another chance but I’m not a massive fan of baby groups. I do them but I don’t particularly socialise. I’m like off you go, they’ll play and I’ll just sit. So for me it was a chance to meet other mums as well. That was a big draw. (Parent)*  *I think probably as I said before, group size, like mindedness, I think just the relaxed atmosphere. Totally relaxed. And if we were talking amongst ourselves… not amongst ourselves like, you know… but if… if the members of the group were having a good discussion, the facilitators sort of chipped in but they didn’t try and steer it in any way, you know, they’d just let that flow. And I think that was quite important, because we weren’t into this sort of rigid situation. (Parent)* |
| **Feasibility** | | |
| Nature of programme content and delivery | Baby versus Toddler programmes  Compatibility with policy initiatives | *“So a discussion was with one of the advisors for the Children’s Centres and how they can be using that in their offer, so, you know, I think pretty keen to be running Incredible Babies. I suppose the… the only reservation we currently have is with Incredible Toddlers and just the practical considerations and the financial considerations about providing childcare, which…I think it’s become apparent that it’s really necessary to do, but, you know… and it’s… they showed us the benefits of doing it, but how much of that we will be able to… to manage from a practical point of view.”* (Service manager)  *“Yeah, this was quite a bone of contention for the health colleagues in that they were very concerned about the bottles in some of the vignettes.”* (Leader)  *"When you were actually going through some of the vignettes and stuff like that it was there, we'd be look at some of vignettes and going, ooh, can't really say that can you, it's like that doesn't fit in with baby-friendly particularly, I think it was the baby one wasn't it? It was… Yeah. Use of bottles or something”* (Leader) |
| Flexibility of service | E-SEE Steps as a development opportunity  Infrastructure – staff roles and levels  IY accreditation process | *“Well, we wouldn’t have been able to access the training. That’s come with this piece of research, training made available to our service, so we wouldn’t have had that opportunity to find that funding to provide that and we would definitely not have been able to offer the clinical supervision that we provided in the early stages. So we knew it was something that was right but we didn’t have that research to tap into to make that happen*.*”* (Service manager)  *“…it was a real incentive to think we can train… get some of our workforce trained, and we can also deliver this to the population, and… is this something we can harness going forward so that we carry on using those skills that we’ve been taught, and be able to deliver future groups* (Service manager)  *So I think all I’m trying to say is, it wouldn’t [be feasible]…we are the 0-5 service. We’re an expensive service…And I think that is the… where the issues would lie. So I think we could do it, if we did it with our staff that…”*  Interviewer: “The Band 5?”  *“Yeah, where their time isn’t so pressured and cost… and costly. But certainly our Band 5 staff would have the flexibility and… to be able to commit to those kinds of programs more than our Band 6s*.*”* (Service manager)  *“…the usual thing that you see on training is some very strong participants who just seem to get it, and their experience lends themselves to… to kind of understanding the programme quickly and being comfortable with role-plays and that kind of thing. And then you get participants who really didn’t, or don’t quite understand what was expected of them, and it’s just not a good fit*.*”* (IY mentor)  *“I really, really would like to…But again it comes down to budget and if I'm going to be here in six months.”* (Leaders)  *“I think I was looking into getting accredited but I think because it… you know, with our organisation, I just don't think… and I know my manager had looked into it as well to see whether it would be funded by our organisation but I think at the moment, because we're just so… the uncertainty is… you know, we just don't know what's going to be happening moving forward that I just don't think for me it's an option at the moment.”* (Leaders) |
| System fit |  | *“Where they have stopped doing other things and they have focused on that [Incredible Years], which is brilliant. So we look at my area as perinatal mental health child mental health is part of that. There is nothing like that in our area at all, so I try and kind of talk to anyone I can about referring in because it is such a good opportunity for families, and I am really, really happy that it is continuing*.*”* (Leaders)  *“I think the difficulty probably was a slight underestimation of the demand on our service, but in conjunction with that, we were going through a huge period of change.”* (Service manager)  *“The problem is as an organisation we would probably like to do it and support it, but we're very, very constrained by the commissioning arrangements.”* (Service manager)  *“…the problem has been in capacity again. The asset we've had, which is very, very… it is really down to one person, is that the practitioner who has delivered the program in the [site] area absolutely thinks it's wonderful… and so I've just given her the go ahead to go and do it. But actually, for her and me, we are slightly going against the grain, because she has just found it so useful, and she says the families she has worked with have been really… she is seeing differences in them. But we are not getting that message from higher up.”* (Service manager)  *“The biggest grievance, if you like, that people had about it was how it was going to work in partnership with Health because immediately we got told about it Health were saying, “Oh, we can’t do that. We can’t do that.” There was always we seemed to be the one having to try and push it. There was a real mixture actually. We’re used to… to projects being just dropped in our… our doorstep with no noticed, so…and I have to say the partnership working with Health there’s been quite a lot of negativity from Health as well, “We haven’t got time to do this. It’s not our… our priority.” That’s been the… that’s been by far the biggest, biggest challenge.”* (Service manager)  *“It’s something… yeah, it’s like… so… it used to be delivered, and I would really like to see that back within the Family Hubs being delivered again…But we are now with the 0-5 service, and I don’t… I don’t know from a budget point of view how it would work…And that… yeah, and I think that’s just the crux of it, money!”* (Service manager) |
