# Supplementary material for: Barriers and facilitators in the delivery of a proportionate universal parenting program model (E-SEE Steps) in community family services
Source: PLoS One. 2022 Jun 13;17(6):e0265946. doi: 10.1371/journal.pone.0265946 (PMC9191704; doi:10.1371/journal.pone.0265946)
Supplement: S4 Table — Practitioner characteristics (A) and practitioner confidence in setting up and running a parenting group (B). (DOCX) [file pone.0265946.s006.docx]

**S4A Table. Practitioner characteristics**

| Practitioner characteristic | | n | Percentage (%) |
| --- | --- | --- | --- |
| Gender | Female | 50 | 100 |
|  | Male | 0 | 0 |
| Ethnicity | White British | 43 | 86 |
|  | British ‘other’ | 7 | 14 |
| Qualifications | Undergraduate degree | 21 | 42 |
|  | Level 3 (QCF/NVQ/NNEB/A level) | 13 | 26 |
|  | BTEC | 6 | 12 |
|  | Pre-graduate diploma | 5 | 10 |
|  | Post-graduate diploma | 4 | 8 |
|  | Master’s degree | 1 | 2 |
| Role* | Health visitor (Grade 6) | 5 | 10 |
|  | Community Health Nurse (Band 5) | 2 | 4 |
|  | Assistant Health Visitor (Band 4) | 3 | 6 |
|  | Trainee Nursing Associate | 1 | 2 |
|  | Mental Health Practitioner (Band 6) | 1 | 2 |
|  | Area Parenting Coordinator (Grade 5) | 4 | 8 |
|  | Family Support Worker (Grade F/4) | 22 | 44 |
|  | Senior Early Years Worker (Grade E/3) | 3 | 6 |
|  | Children and Families Support Worker (Band 3) | 4 | 8 |
|  | Healthy Child Practitioner | 2 | 4 |

*missing data n=3

**S4B Table. Practitioner confidence in setting up and running a parenting group**

|  | **Trained only  n = 24 (%)** | **Delivered  n = 26 (%)** |
| --- | --- | --- |
| > 50% of direct work is with parents of young children | 96% | 85% |
| Percentage receiving clinic practice supervision | 92% | 89% |
| Percentage of part-time staff | 63% | 27% |
| Percentage who had previously delivered parenting programs | 54% | 65% |
| Percentage who had delivered a parenting program in the last 5 years | 42% | 65% |
| Percentage who had previously delivered an IY program | 13% | 27% |
| Reported feeling ‘confident’ in setting up and running a parenting group *pre-training* | 29% | 42% |
| Reported feeling ‘confident’ in setting up and running a parenting group *post-delivery* | - | 59% |
| Reported feeling supported by their organization during delivery of the IY program | - | 80% |
